# Preparation of coal gangue ceramsite high-strength concrete and investigation of its physico-mechanical properties

**DOI:** 10.1038/s41598-022-20940-y

**Published:** 2022-09-30

**Authors:** Hongbo Guan, Jitao Yu, Albert Salomon Umuhuza Kibugenza, Qingwei Sun

**Affiliations:** School of Civil Engineering, Liaoning Technical University, Fuxin, 123000 China

**Keywords:** Civil engineering, Structural materials

## Abstract

Using coal gangue (CG) as a building material does not only reduce the disposal of industrial waste and promote the resource utilization of solid waste, but also solves the excessive consumption of sand and stone in construction. This study experimentally investigated calcining ceramisites from CG raw materials and the mechanical properties of CG ceramsite concrete were studied. Additionally, the physical, chemical and composition changes of CG before and after calcination were observed using scanning electron microscopy and X-ray diffraction analysis (XRD). The experimental results reveal that calcination can reduce the density, increase the strength, increase the porosity of CG, and change the microstructure and mineral composition of CG. Finally, there are great differences between coal gangue ceramsite concrete and ordinary concrete in the variation of compressive strength with time and the relationship between elastic modulus and compressive strength. In this paper, the existing formula is modified according to the experimental data.

## Introduction

Coal gangue (CG) is a type of industrial solid waste produced by the process of coal excavation and separation^1–3^. Generally, one ton of CG is discarded for every 10 tons of produced coal^4,5^. Statistics show that 5–6 billion tons of CG are now stored, and the accumulation increases at the rate of 150–200 million tons per year in China^6,7^. Presently, most CG is disposed by simple stacking, and there are approximately 2600 large-scale CG hills in China, which amount to approximately 15,000 hectares^8–10^. This does not only result in resource wastage, but also causes environmental pollution and threatens the health and well being of local communities^11^. With the implementation of China’s green and sustainable development strategy, the rational and comprehensive utilization of CG will bring remarkable economic, environmental, and social benefits.

Existing research on the main application methods of CG in building materials includes research on the production of cement, burning bricks, concrete hollow blocks, and aerated concrete^12,13^. Although there are different types of CG with different properties owing to the different origins of CG, most chemical and mineral components are similar to natural aggregates (NAs). Therefore, a more direct and effective method of using CG is to use it as a coarse or fine aggregate in concrete after crushing^14–16^. However, CG aggregates (CGAs) have looser structure and exhibit lower physical properties compared with NAs. Therefore, the strength of concrete with CG as the aggregate is lower than that of concrete with NA as the aggregate under the same mix proportion^17–19^. Therefore, to improve the mechanical properties of CGA concrete and enable its adoption in more construction fields, it is necessary to improve the physical and mechanical properties of CGA.

Although the chemical composition of CG is complex, CG is mainly composed of silicon and aluminum, and contains more than a dozen elements. Generally, CG is mainly composed of oxides, such as SiO_2_, Al_2_O_3_, Fe_2_O_3_, CaO, MgO, NaO, and K_2_O^20–22^. Calcined CG is an effective method for improving the aggregate properties. Carbon and various other components in CG can be removed during calcination at the temperature range of 500–800 °C, and the kaolinite in CG can also be gradually transformed into metakaolin^23,24^. Zhang et al.^25^, Cao et al.^13^, and Guo et al.^26^ pointed out that CG has high activity after calcination at the temperature of 700–800 °C. The secondary hydration reaction of metakaolin and cement hydration products (calcium hydroxide) can improve the mechanical properties of CGA concrete. Yang et al.^27^ found that high temperature calcination can cause the internal chemical reaction of CG, eliminate unstable components in CG, generate stable substances, and cause corresponding changes to the physical properties of CG. By calcination, CGA can be converted into a lightweight and high-strength ceramsite aggregate^5,28^. Compared with ordinary concrete, ceramsite lightweight aggregate concrete has excellent properties, such as low density, high cylinder compressive strength, high porosity, high softening coefficient, good frost resistance, and excellent alkali-aggregate resistance^29–31^. Many studies^32–36^ have investigated the preparation and performance of ceramsite concrete with the objective of further improving its performance. To improve the performance of CG ceramsite lightweight aggregate concrete (CGCLAC), it is necessary to improve the physical and mechanical properties of coal gangue ceramsites. However, research on the preparation of high-strength ceramsite from CG raw materials is still relatively rare, and studies on the constitutive properties of CGCLAC are even fewer.

This study experimentally investigated the production of calcining ceramisite from CG raw materials. The raw material formula and experimental process used in this study can be used as the preliminary basis for the experimental investigation of CGC. The physical, chemical, and composition changes of CG before and after calcination were observed by scanning electron microscopy (SEM) and X-ray diffraction analysis (XRD). Coal gangue ceramsite (CGC) made by this method has high strength. Moreover, this study conducted mechanical tests on concrete prepared using CGC. The variation law of strength is explored and the relationship between elastic modulus and compressive strength is put forward.

## Preparation of coal gangue ceramsite

### Raw materials

According to Riley’s results^37^ obtained by the investigation of calcined ceramsite, the chemical composition of raw materials that is appropriate for the production of ceramsite is presented in Table 1. The SiO_2_ and Al_2_O_3_ form glass melt at high temperature, and their interaction in the liquid phase promotes the formation and growth of 3Al_2_O_3_·2SiO_2_. If the SiO_2_ and Al_2_O_3_ content in the raw material increases, the melting temperature increases, the viscosity of the liquid phase increases, the expansion becomes lower, and the strength of the raw material becomes greater. With a higher content of SiO_2_ and Al_2_O_3_ in ceramsite raw materials, higher temperature is required to reach a certain viscosity. The K_2_O, Na_2_O, CaO, MgO, and so on, are cosolvent, which is beneficial for reducing the melting point of raw materials. For example, when SiO_2_ and Al_2_O_3_ generate eutectic compounds, the melting temperature is 1713 °C; when K_2_O is added, the melting temperature is 976 °C; when Na_2_O is added, the melting temperature is 874 °C. At high temperature, Fe_2_O_3_ and C produce gas substances such as H_2_O, CO, CO_2_, and other gas substances, which are the force driving the pore expansion of ceramsite.

**Table 1:** Chemical composition appropriate for the production of ceramsite (%).

Chemical composition	SiO_2_	Al_2_O_3_	Fe_2_O_3_	CaO + MgO	K_2_O + Na_2_O
Suitable scope	48–70	8–25	3–12	1–12	0.5–7
Ideal scope	60–70	15–25	5–10	0–5	3–4

The data in the table is obtained by XRD (Ultima IV, Rigaku, Japan).

The main components of CG are clay minerals, mainly kaolinite and hydromica, and also quartz, feldspar, pyrite, carbonate, and other secondary minerals. Table 2 presents the chemical composition of CG in Fuxin, Liaoning, China. By comparing Tables 1 and 2, it can be found that CG is an ideal raw material for the production of calcining ceramsite. Even if some chemical components do not satisfy the standard, the content of raw materials can be adjusted by adding other substances to reach the ideal range.

**Table 2:** Chemical composition of CG (%).

Chemical composition	SiO_2_	Al_2_O_3_	Fe_2_O_3_	CaO	MgO	Na_2_O	K_2_O	SO_2_	TiO_2_	IL
Coal gangue	65.36	22.85	5.69	0.95	0.80	0.18	2.96	0.56	1.39	23.21

The data in the table is obtained by XRD (Ultima IV, Rigaku, Japan).

### Calcination process of CGC

The production process of CGC includes raw material processing, granulation, and thermal processing. The CG was broken by a jaw crusher and milled by a ball mill. The raw material was screened using a 100 mesh sieve. Various raw materials were mixed according to a certain ratio to prepare pellets with a diameter of 10–20 mm. Then, the formed CGC was put into a drying box and heated to 105 °C for 1–2 h. Preheating was carried out at 300 °C for 30 min to further remove the surface moisture so as to eliminate surface cracking and bursting of CG in the calcination process caused by a sharp increase in temperature. The preheating and calcination of the sample were carried out in a preheating furnace and calcination furnace, respectively. When the CG raw material reached the preheating time at the design preheating temperature, it was immediately removed from the preheating furnace and put into the calcination furnace, which had reached the design calcination temperature of 1150 °C, and the calcination time was 30 min. The rapid cooling method was used to quickly decrease the surface temperature of ceramsite below 400 °C. When the ceramic was calcined, as the temperature changed, the internal material composition of the ball resulted in the following reaction process:

1. Chemical reaction at 400–800 °C:
  $$ {\text{C}} + {\text{O}}_{{2}} \to {\text{CO}}_{{2}}{\uparrow}\quad {\text{2C}} + {\text{O}}_{{2}} \to {\text{2CO}}{\uparrow}\quad {\text{CO}}_{{2}} + {\text{C}} \to {\text{2CO}}{\uparrow}$$
  $$ {\text{Al}}_{{2}} {\text{O}}_{{3}} \cdot{\text{2Si}}_{{2}} {\text{O}}\cdot{\text{2H}}_{{2}} {\text{O}} \to {\text{Al}}_{{2}} {\text{O}}_{{3}} \cdot{\text{2Si}}_{{2}} {\text{O}} + {\text{2H}}_{{2}} {\text{O}}{\uparrow}\quad {\text{MgCO}}_{{3}} \xrightarrow{{400 \sim 500\;^\circ {\text{C}}}} {\text{MgO}} + {\text{CO}}_{{2}}{\uparrow}$$
2. Chemical reactions at 800–1100 °C:

$$ {\text{2FeS}} + {\text{3O}}_{{2}} \to {\text{2FeO}} + {\text{2SO}}_{{2}}{\uparrow}\quad {\text{2Fe}}_{{2}} {\text{O}}_{{3}} + {\text{C}} \to {\text{4FeO}} + {\text{CO}}_{{2}}{\uparrow}\quad {\text{2Fe}}_{{2}} {\text{O}}_{{3}} + {\text{3C}} \to {\text{4Fe}} + {\text{3CO}}_{{2}}{\uparrow}$$

$$ {\text{Fe}}_{{2}} {\text{O}}_{{3}} + {\text{C}} \to {\text{2FeO}} + {\text{CO}}{\uparrow}\quad {\text{2Fe}}_{{2}} {\text{O}}_{{3}} + {\text{2C}} \to {\text{4FeO}} + {\text{2CO}}{\uparrow}\quad {\text{S}} + {\text{O}}_{{2}} \to {\text{SO}}_{{2}}{\uparrow}$$

$$ {\text{Al}}_{{2}} {\text{O}}_{{3}} \cdot{\text{2Si}}_{{2}} {\text{O}} \to {\text{Al}}_{{2}} {\text{O}}_{{3}} + {\text{2Si}}_{{2}} {\text{O}}\quad {\text{CaCO}}_{{3}} \xrightarrow{{800 \sim 900\;^\circ {\text{C}}}} {\text{CaO}} + {\text{CO}}_{{2}}{\uparrow}\quad {\text{FeS}}_{{2}} \xrightarrow{900^\circ } {\text{FS}} + {\text{S}} $$

$$ {\text{4FeS}}_{{2}} + {\text{11O}}_{{2}} \xrightarrow{{1000 \pm 50\;^\circ {\text{C}}}} {\text{2Fe}}_{{2}} {\text{O}}_{{3}} + {\text{8SO}}_{{2}}{\uparrow}$$

$$ {\text{CaCO}}_{{3}} + {\text{SiO}}_{{2}} \xrightarrow{{1000\;^\circ {\text{C}}}} {\text{CaSiO}}_{{3}} + {\text{CO}}_{{2}}{\uparrow}$$

$$ {\text{2CaSO}}_{{4}} \xrightarrow{{1100\;^\circ {\text{C}}}} 2{\text{CaO}} + {\text{2SO}}_{{2}}{\uparrow}+ {\text{O}}_{{2}}{\uparrow}$$

The above reactions indicate that the combustion of elements such as S and C form CO_2_ and SO_2_ gas overflow, and the carbonate compounds (CaCO_3_ and MgCO_3_) and sulfides are thermally decomposed. In the calcination process, various unstable substances are gradually decomposed and discharged, and the remaining substances are not easy to decompose, which results in the more stable properties of ceramsite.

### Changes of physical and mechanical properties after calcination

**Figure 1:**
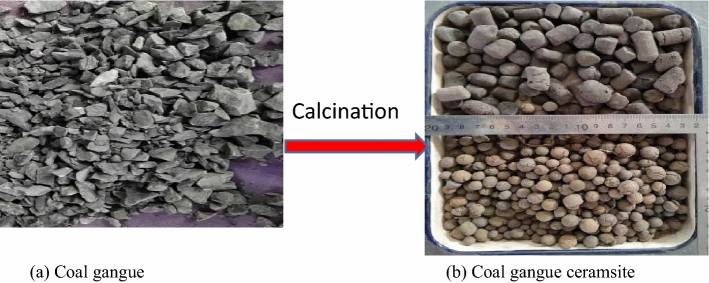
Change of ma croscopic morphologies.

**Figure 2:**
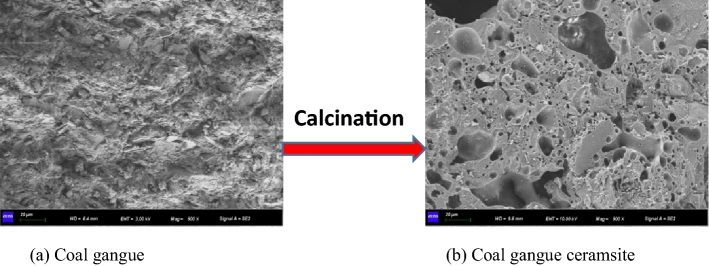
Change of microscopic morphologies.

The macroscopic and microscopic morphologies of CG before and after calcination are shown in Figs. 1 and 2, respectively. The CG is black or black gray; however, according to the difference of the chemical composition and calcination temperature, the ceramic particles exhibit different colors, such as white, gray, iron red, and earth yellow, after calcination. As can be seen in the SEM images, the surface of the CG ceramsite is in a molten state, its surface distribution is approximately 30 μm pores, and many pores are distributed within it. The internal pores can essentially be divided into circular macropores with a diameter of 50–200 μm between the particle skeleton, and small pores with diameter of less than 30 μm on the skeleton. In the high-temperature calcination process, the iron oxide and carbon in the ceramsite undergo a redox reaction and release high amounts of CO and CO_2_. These gases are bound by the liquid phase formed by the matrix, which results in the formation of voids during the expansion of the ceramsite volume. The amount of expansive gas and the uniformity of pores are not only related to CG, but are also directly related to the calcination temperature. When the surface produces a higher amount of viscous liquid, the amount of bound gas and the pores in the ceramsite increase, and the uniformity improves, which decreases the density of ceramsite while increasing its strength.

According to the testing methods for lightweight aggregates specified in *Part 2: Test methods for lightweight aggregates* (GB/T17431.2-2010)^38^, the physical and mechanical properties of CG should be tested before and after calcination. The performance indicators are listed in Table 3. After calcination, the physical and mechanical properties of CG changed substantially. The apparent density, loose bulk density, and tap bulk density decreased by 28.11%, 40.47%, and 45.58%, respectively, while the void ratio and water absorption increased by 30.12% and 79.86%, respectively, and the crushing index decreased by 29.49%.

**Table 3:** Basic properties.

Type	Apparent density (kg m^−3^)	Loose density (kg m^−3^)	Tap density (kg m^−3^)	Void ratio (%)	Water absorption (%)	Crushed value (%)
CG	2610	1490	1680	40.5	4.17 (1 h)	23.4
CGC	1876.2	887	914.3	52.7	7.5 (1 h)	16.5

The quality of the CGC was evaluated based on the *Technical standard for application of lightweight aggregate concrete* (JGJ/T12-2019)^39^ and *Pebble and crushed stone for construction* (GB/T14685-2011)^40^. The CG ceramsite satisfies the requirements of Class II coarse aggregates, while CG only satisfies the requirements of Class III coarse aggregates. The grade of CG used in construction can be improved by calcining.

### Changes of chemical constituents and mineral composition after calcination

**Figure 3:**
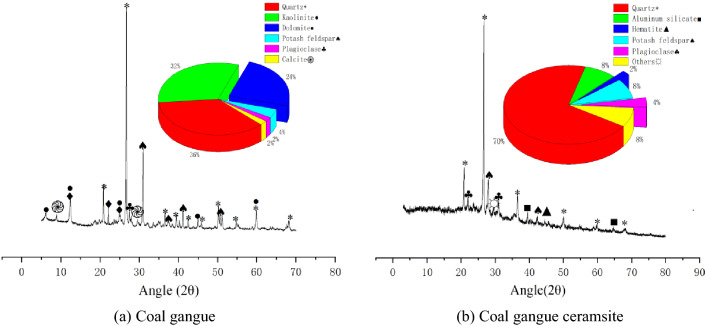
XRD spectra of coal gangue before and after calcination.

The mineral composition of CG before and after Calcination was determined by XRD (Ultima IV, Rigaku, Japan), according to *the specification of the X-ray diffractometer* (JB/T 9400-2010)^41^ and *the analysis method for clay minerals and ordinary non-clay minerals in sedimentary rocks using X-ray diffraction* (SY/T 5163-2018)^42^. A quantitative analysis scanning method, namely, step scanning, was used in this test; the sample interval and scanning speed were set to 0.01 and 0.25 /min, respectively. The XRD test results are shown in Fig. 3.

Quartz and kaolinite are the two major crystallized minerals in the uncalcined CG. Quartz is hard and wear resistant, which contributes to the high strength of CG, while kaolinite is a hydrous layer silicate clay mineral and a type of soil or lump with low hardness and poor stability, which is one of the reasons for CG being easy to break and having low strength. The CG contains easily-hydrolyzed and weathered chemical components, which account for approximately 15%. After calcination, the loss of original materials in CG greatly increases the proportion of quartz, which contributes to the improvement of the mechanical properties of CGC. The alumina silicate in kaolinite is calcined at high temperature to form mullite, which is a material with high strength that further improves the mechanical properties of calcined CGC. Additionally, with high temperature calcination, the unstable components in CG are transformed into stable substances in CGC. Therefore the physical and mechanical properties of CGC are qualitatively different to those of CG before calcination.

## Testing of physico-mechanical properties

### Preparation of CGCLAC specimens

In this test, 42.5R ordinary Portland cement was used as the concrete cementitious material, and an appropriate amount of fly ash was added. Local river sand was used as the fine aggregate of concrete with a fineness modulus of 3.26. The CGC with the particle size ranging from 5 to 20 mm was used as a coarse aggregate, and the water for mixing was ordinary tap water. A water reducing agent was added to the fresh concrete to ensure that the concrete slump satisfies the concrete mixing requirements. The water reducing agent dosage was 1.5–2.5% of the cement dosage, and the water reducing rate was 20–30%.

Four different strength mix proportions were designed according to the needs of different engineering practices. The mix proportions of CGCLAC are presented in Table 4. The compressive strength test, splitting tensile strength test, flexural strength test, and elastic modulus test of each mixture ratio were completed. The compressive strength and the splitting tensile strength were tested using a cubic test block with the size of 100 mm × 100 mm × 100 mm, the flexural strength was determined using prismatic specimens with a size of 100 mm × 100 mm × 550 mm, and the elastic modulus was determined using prismatic specimens with a size of 100 mm × 100 mm × 300 mm. The compressive strength was tested at the curing age of 3 days, 7 days, 14 days, 21 days, and 28 days, respectively. The splitting tensile strength, flexural strength, and elastic modulus were tested only at the curing age of 28 days. Each strength value represents the average strength of three test blocks.

**Table 4:** Mixed proportions of CGCLAC (kg m^−3^).

Type	Materials							
	Cement	CGC	Sand	Water	Fly ash	Additional water	Water reducing agent	W/C
A	300	770	770	165	30	46.20	6.00	0.55
B	360	770	770	162	36	46.20	7.20	0.45
C	432	770	770	151	43	46.20	8.64	0.35
D	475	770	770	143	48	46.20	9.5	0.30

The quantity of each raw material in concrete was calculated according to the mix proportion. According to a previous study, it is necessary to prewet CGC to avoid the difference in the fluidity of concrete caused by excessive water absorption in the mixing process of CGC. The general practice is to add 60% of water absorbed by CGC in one hour into the CGC before making the CGCLAC, such that the CGC can be fully wetted^43^. A water reducing agent was added to the stirring water. Next, all CGCs, sand, and 70% water were mixed in a mixer for 10–20 s, and the cement and fly ash were then added over 30 s; the remaining 30% of water was added over 60 s. When the concrete was uniformly stirred, it was poured into the prepared mold, and then vibrated on a vibration table to be made dense. After 24 h, the mold was removed and the concrete was placed into a standard incubator with a temperature of 20 °C ± 2 °C and a relative humidity of 95% to cure for 28 days.

### Testing of CGCLAC specimens

**Figure 4:**
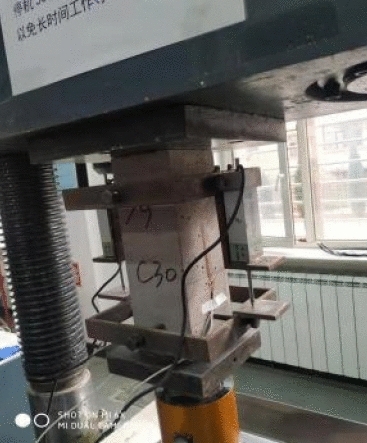
Test device and loading diagram.

**Figure 5:**
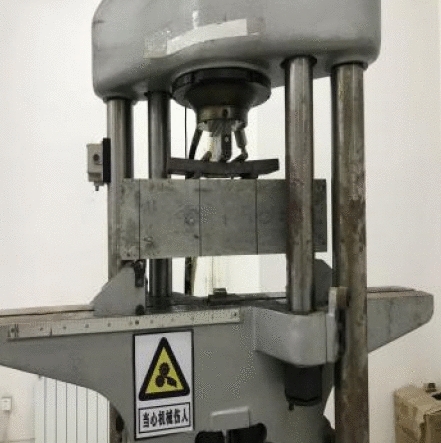
Loading device for four-point flexure test of concrete.

The mechanical properties of CGCLAC were tested according to *Standard for test methods of concrete physical and mechanical properties* (GB/T 50081-2019)^44^, Standard for test method performance on ordinary fresh concrete(GB/T 50080-2016)^45^ and *Technical standard for application of lightweight aggregate concrete* (JGJ/T 12- 2019)^39^. The compressive strength, split tensile strength and elastic modulus were measured using a 200-ton electro-hydraulic servo hydraulic testing machine. The test device is shown in Fig. 4. The flexural tensile strength was measured using a 60-ton electro-hydraulic servo hydraulic testing machine. The loading device used in the test is shown in Fig. 5. The entire loading process was controlled by stress. The loading rate of the compressive strength test was 0.5 MPa/s, and the loading rate of the splitting tensile strength and flexural tensile strength was 0.05 MPa/s. The test data were automatically recorded by a data-acquisition instrument, and the data acquisition frequency was 0.2 s.

According to *General rules for measurement of length in micron scale by SEM* (GB/T16594-2008)^46^, after the mechanical test, a test block with a surface area of up to 1 cm^2^ and thickness up to 1 cm was drilled from the surface of the damaged specimen using a hollow drill. The specimens were pumped into a vacuum for SEM analysis, and the microstructure of the failed specimen was observed using a scanning electron microscope (TESCAN MIRA4, Czech Republic).

## Results and discussion

### Fresh concrete properties

The slump mean values of series A, B, C, and D were 69 mm, 65 mm, 53 mm, and 42 mm, respectively. Additionally, the cohesion and water retention of concrete were satisfactory. The test results reveal that the slump satisfies the requirements of concrete construction specified in *Ready-mixed concrete* (GB/T14902-2012)^47^. The cohesion of cement slurry mainly depends on the dry-thin degree of cement slurry, that is, on the consistency of cement slurry, and the frictional resistance between the aggregates, mainly depends on the thickness of the cement slurry layer on the surface of the aggregates, that is, on the quantity of cement slurry. Considering the performance difference between the CGC and the natural aggregate, the traditional concrete mix design method considers that the strength increases as the sand ratio decreases, which obviously does not satisfy the requirements of the CGCLAC mixture. Therefore, in CGCLAC mix design under different conditions and the same circumstances, the selected sand ratio is larger compared with that of ordinary concrete.

### Water absorption and concrete density

Table 5 presents the experimental results obtained for the apparent density and water absorption of different concrete mixtures. As can be seen, the CGC concrete densities are lower than that of ordinary concrete. This essentially satisfies the requirements of lightweight aggregate concrete, which typically has an apparent density < 1950 kg/m^3^. The main reason for this is the fact that, in this study, the apparent density of CG was 1876.2 kg/m^3^, and the apparent density of NA was mostly above 2600 kg/m^3^. Therefore, CGC concrete is much lighter than NA concrete.

**Table 5:** Density and water absorption of hardened concrete.

Type	Apparent density (kg/m^3^)	Water absorption (%)
A	1975.4	3.43
B	1977.8	3.36
C	1980.3	3.32
D	1984.7	3.30

The water absorption of CGCLAC is much higher than that of ordinary concrete (the water absorption of ordinary concrete is between 2 and 3%). Both the density and absorption are closely linked to the characteristics of the used aggregate. The CGC aggregates are less dense and more absorbent compared with natural aggregates. The reason for this is the existence of many pores on the surface of the CGC aggregate. This results in more mortar being attached to the aggregate surface, which in turn results in the CGC concrete having lower density and higher absorption rate.

### Compressive strength

**Figure 6:**
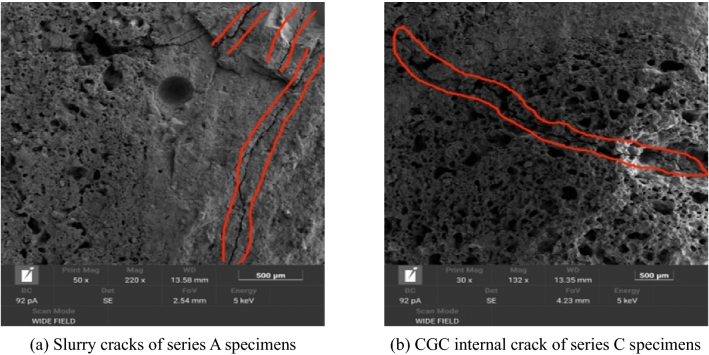
Crack development under failure.

The compressive strength of concrete is an important index for determining the strength grade of concrete. The test results reveal that, the compressive strength steadily increased as the water–cement ratio of concrete decreased, similar to ordinary concrete. The compressive strength of concrete with a water–cement ratio 0.30 is slightly lower than that of concrete with a water–cement ratio of 0.35. As shown in Fig. 6, as the compressive strength increased, the main reason for the damage gradually changed from the damage of the slurry and interface to the damage of the aggregate. Therefore, reducing the water–cement ratio does not improve the compressive strength of concrete.

**Figure 7:**
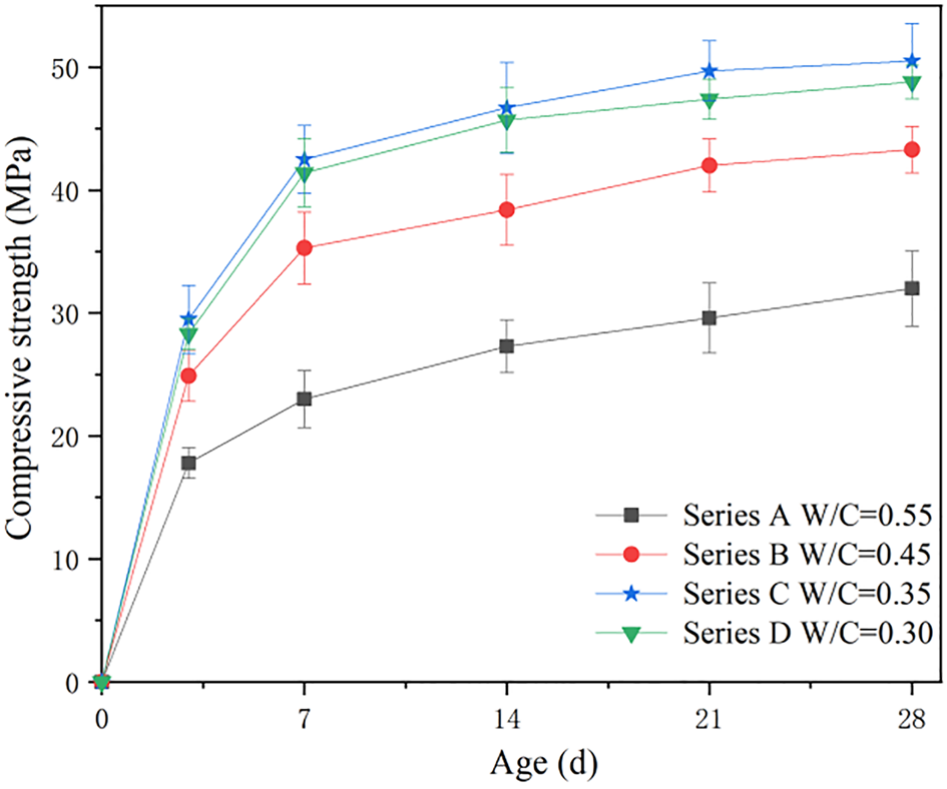
Relationship between compressive strength and age of cube.

The cube compressive strength of CGCLAC was measured at 3 days, 7 days, 14 days, 21 days, and 28 days, and the results are presented in Table 6. The variation curve of the cubic compressive strength of CGCLAC with the curing age is plotted in Fig. 7.

**Table 6:** Cube compressive strength of coal gangue ceramsite coarse aggregate concrete.

Series	W/C	Cube crushing strength (MPa)/Coefficient of concrete age (%)				
		3d	7d	14d	21d	28d
A	0.55	17.81/55.97	23.05/72.33	27.35/85.85	29.60/93.08	31.82/100
B	0.45	24.92/57.51	35.34/81.52	38.44/88.68	41.92/96.77	43.33/100
C	0.35	29.54/58.42	42.52/84.16	46.73/92.48	49.73/98.42	50.53/100
D	0.30	29.33/60.04	41.43/84.84	45.72/93.65	47.45/97.13	48.82/100

(1) Each strength is the average value of three test specimens.

(2) The coefficient of concrete age is the ratio of the compressive strength of concrete at different ages to that at 28 days.

From Table 6 and Fig. 7, it can be seen that the early strength of CGCLAC developed rapidly, reaching more than 55% of the 28-day compressive strength in three days, and more than 80% of the 28-day compressive strength in seven days, except for series A. This growth rate is significantly higher than that of ordinary concrete. The 3-day compressive strength of ordinary concrete is generally close to 50% of the 28-day compressive strength, while the 7-day compressive strength is typically close to 70% of the 28-day compressive strength. After more than 14 days, the compressive strength of CGCLAC develops slowly, mainly owing to the great difference between the physical and mechanical properties of the CGC aggregate and natural aggregate.

In concrete engineering, the 28-day compressive strength of concrete under standard curing conditions is generally considered as an important parameter in the acceptance evaluation of unit engineering quality. The 28-day compressive strength can be predicted by the early compressive strength of concrete, which provides the basis for further construction. Therefore, it is necessary to investigate the relationship between the age and compressive strength of CGCLAC. According to the existing empirical formula for ordinary concrete, the formula describing the relationship between the early compressive strength and 28-day strength of CGCLAC is expressed as follows:

1 $$ \frac{{f_{cn} }}{{f_{c28} }} = a\frac{lg(n)}{{lg\left( {28} \right)}} + b $$

where $f_{cn}$ is the early compressive strength of concrete (MPa); $f_{c28}$ is the 28-day compressive strength of concrete (MPa); *n* is the age (${\text{n}} \ge 3$); *a* and *b* are parameters obtained through regression analysis.

The proposed formula describing the relationship between the early strength and the compressive strength at the standard age was developed using the least squares method and through the regression analysis of the experimental data, as follows:

2 $$ \frac{{f_{cn} }}{{f_{c28} }} = 0.6144\frac{lg\left( n \right)}{{lg\left( {28} \right)}} + 0.{4}0{69} $$

**Figure 8:**
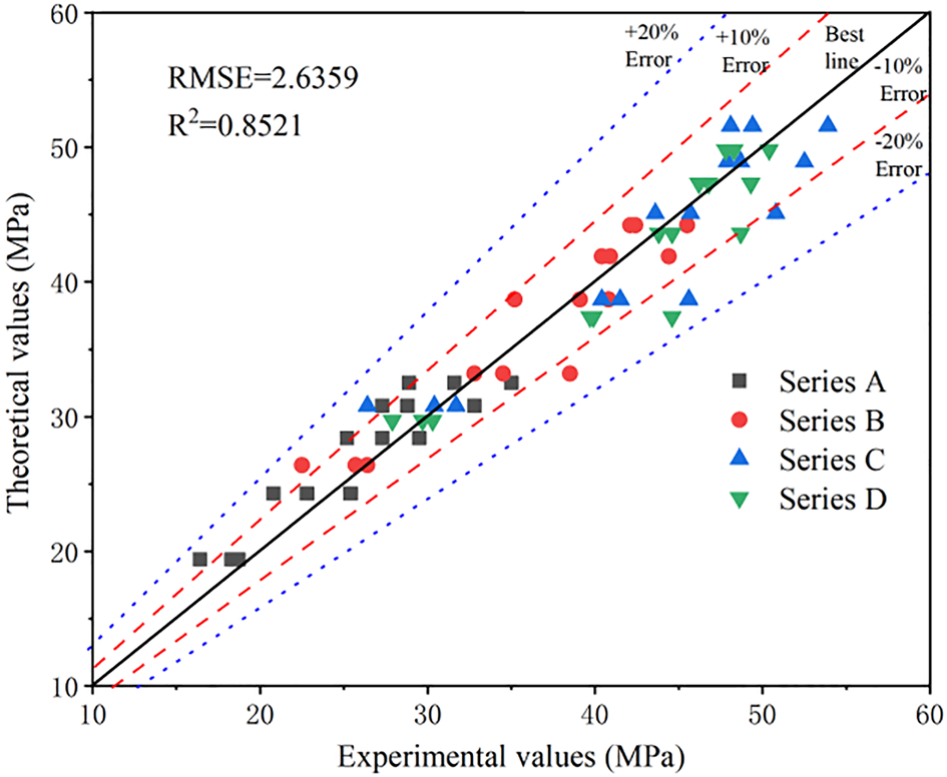
Comparison between theoretical and experimental results for compressive strength of CGCLAC.

Figure 8 shows the values of the compressive strength of CGCLAC versus the experimental values, as obtained by the proposed formula. The preliminary evaluations were carried out using two indices: R^2^ and RMSE. As can be seen, the majority of data fall within the 10% error line, and the maximum error between the calculated and experimental values is 20.3%. The values of R^2^ and RMSE given by this formula are 0.8521 and 2.6359, respectively, which means that the performance of the newly developed formula for estimating the compressive strength is satisfactory. Therefore, the proposed formula can be used in actual projects to predict the compressive strength of CGCLAC at different ages.

### Splitting tensile strength and flexural tensile strength

**Figure 9:**
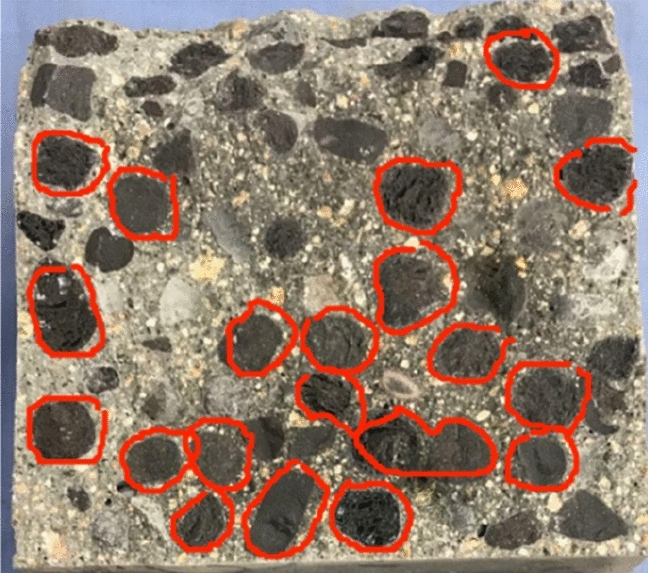
Splitting failure surface.

As a basic mechanical index of concrete, the concrete tensile strength is of great significance to concrete crack resistance. The splitting tensile strength test is a common method for evaluating the tensile strength of concrete. The splitting failure surface of the CGCLAC specimen is shown in Fig. 9. Most of the CGC aggregate was directly split, which is very different to ordinary concrete’s failure surface of cement mortar and interface failure. In fact, the tensile strength of CGCLAC is mainly affected by the strength of mortar, the quality of coarse aggregate, and the bonding performance between the aggregate and the mortar. Table 7 presents the splitting tensile strength test results for the entire mix proportion. The test results reveal that the 28-day splitting tensile strength of CGCLAC is between 2 and 4 MPa, accounting for approximately 7% of the cube compressive strength.

**Table 7:** Test results for main mechanical properties of CGCLAC at 28 days.

Series	W/C	Cube compressive strength (MPa)	Splitting tensile strength (MPa)	Flexural tensile strength (MPa)	Modulus of elasticity (GPa)
A	0.55	31.82	2.24	3.37	18.17
B	0.45	43.33	2.98	4.02	22.50
C	0.35	50.53	3.75	5.33	25.56
D	0.30	48.82	3.65	5.15	24.98

**Figure 10:**
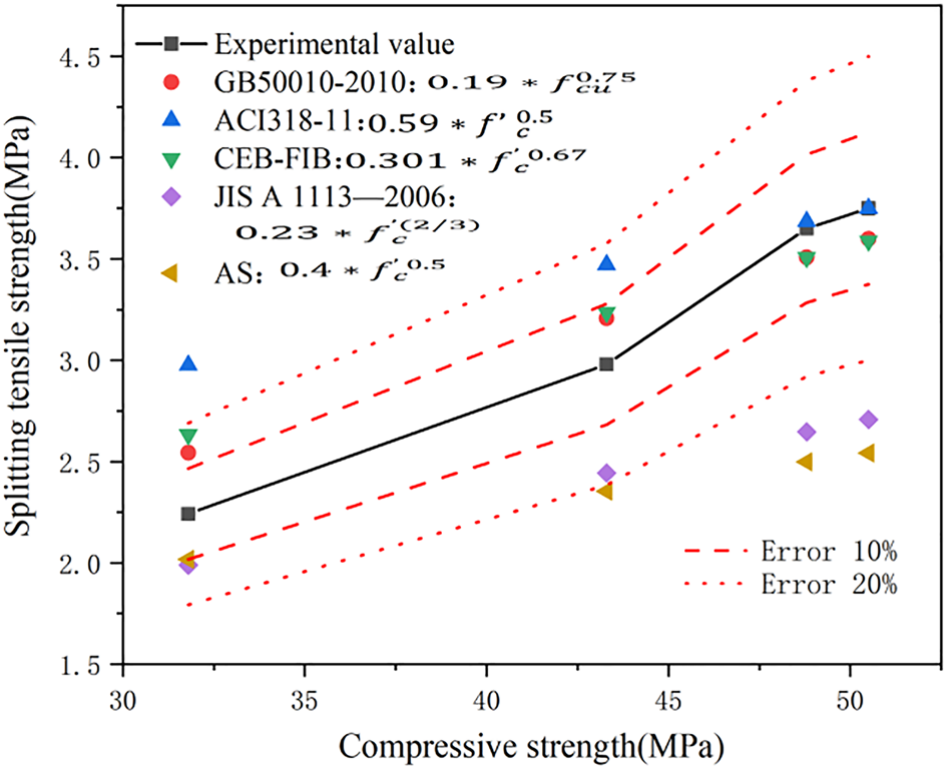
Comparison of predicted splitting tensile strength to experimental value.

Some national codes specify the calculation formula for predicting the tensile strength of concrete according to the compressive strength of concrete. Figure 10 shows the comparison between the experimental and calculated values in GB50010-2020^48^, ACI318-11^49^, CEB-FIB^50^, JIS A 1113-2006^51^, and AS^52^. By carrying out error analysis, the maximum errors between the calculated results and the experimental values in the above-mentioned specifications were determined as 13.5%, 32.7%, 17.4%, 27.8%, and 32.2%, and the RMSE values are 0.2156, 0.4424, 0.2573, 0.7815, and 0.8978, respectively. From the comparison results, it can be concluded that the formulae in the GB50010-2010 and CEB-FIB specifications can satisfactorily predict the splitting tensile strength of CGCLAC.

The experimental data in Table 7 reveal that the 28-day flexural strength of CGCLAC is between 3.3 and 5.5 MPa, while the flexural strength is approximately one tenth of its cube compressive strength. As can be seen, the CGC has good performance in terms of bonding with the cement mortar. Moreover, it can be clearly seen that the CGC is directly damaged at the fracture surface, unlike ordinary concrete.

### Modulus of elasticity

The elastic modulus of concrete is an important performance index in the design and calculation of concrete structures, and directly affects the calculation of the internal force and the deformation of the structure. The elastic modulus of concrete mainly depends on the elastic modulus of the cement mortar and aggregate and their relative content in concrete. Owing to the particularity of the CGCLAC composition, and the difference between the elastic modulus and the deformation performance of the CGC and mortar in the CGCLAC composition, the factors affecting the elastic modulus are more complex compared with those of ordinary concrete. The results obtained for the modulus of elasticity of different concretes are presented in Table 7. According to *Code for design of concrete structures* (GB50010-2020)^48^, when the compressive strength of concrete is 30–50 MPa, the corresponding elastic modulus is 30 GPa to 34.5 GPa. From the results in Table 7, the elastic modulus of CGCLAC is 25%–35% lower than that of ordinary concrete with the same strength.

**Figure 11:**
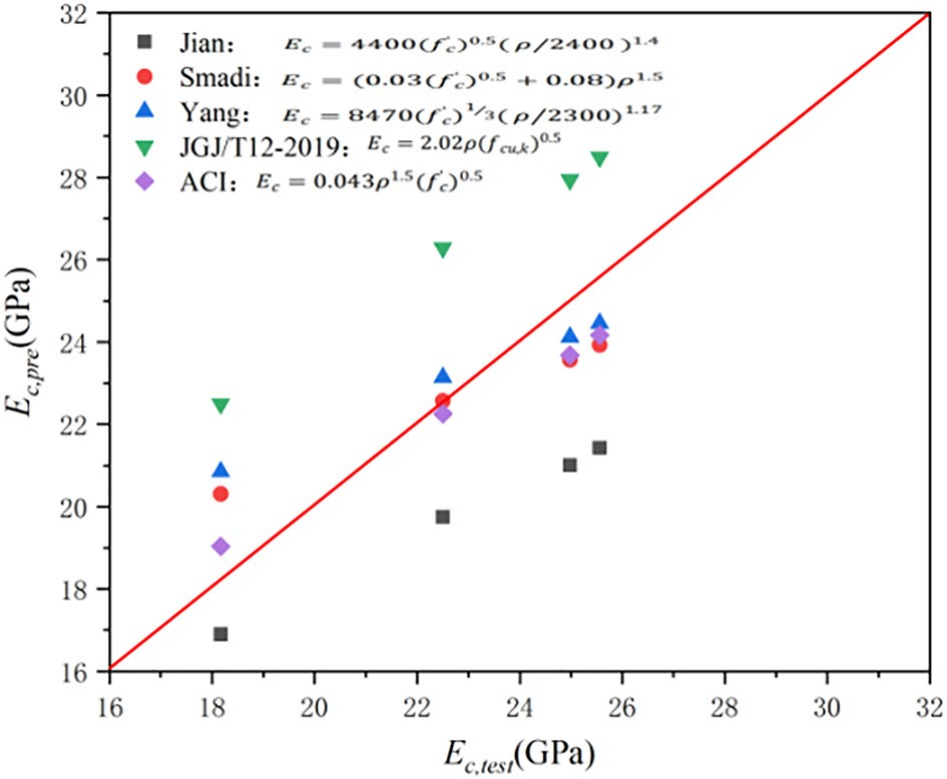
Comparison between calculated and experimental values of elastic modulus.

Based on a large number of theoretical studies and practical cases, the relationship between the compressive strength and elastic modulus of ordinary aggregate concrete and lightweight aggregate concrete was established. The formula for calculating the elastic modulus of the concrete’s compressive strength is provided in the building codes of some countries. The existing research results reveal that the elastic modulus of concrete is a function of the compressive strength and apparent density of concrete. Figure 11 shows the comparison between the experimental values and calculated values obtained by the prediction formulae in the literature. Prediction formulae are mainly based on calculation formulae proposed by various studies and are provided in the building codes of some countries. In Fig. 11, the maximum relative deviations between the experimental values and calculation values of Jian^53^, Smadi^54^, Yang^55^, JGJ/T12-2019^39^, and ACI^49^ are 23.84%, 5.45%, 16.14%, 11.77%, and 14.74%, and the average deviations are 3.5, 0.95, 3.03, 1.31, and 1.32, respectively; the root mean square error (RMSE) is 3.24, 1.52, 1.54, 3.55, and 1.06, respectively. The comparison results reveal that the calculation formula proposed by ACI is more in line with the actual elastic modulus, and can be used to calculate the elastic modulus of ceramsite concrete.

## Conclusions

Coal gangue can be processed into ceramsite concrete by the above method, which solves the problem of solid waste treatment and alleviates the shortage of concrete raw materials. Based on the analysis of test results, the following conclusions were drawn by this study:

1. The physical properties, microstructure, and composition of CG changed after calcination, while the strength and water absorption increased. The apparent density of CGC concrete with different concrete mixes was lower than that of ordinary concrete, which essentially satisfies the requirements of lightweight aggregate concrete.
2. Owing to the great difference between the physical and mechanical properties of CGC aggregate and natural aggregate, the early strength of CGCLAC developed rapidly, mainly because the water absorption of CGC is large and CG ceramsite absorbs a substantial amount of water. Therefore, the hydration reaction between the cement and aggregate is sufficient.
3. Based on the experimental investigation of the cube compressive strength of CGCLAC at different ages, the empirical formula (the following equation) for predicting the early strength of CGCLAC was fitted.
  $$ \frac{{f_{cn} }}{{f_{c28} }} = 0.6144\frac{lg\left( n \right)}{{lg\left( {28} \right)}} + 0.{4}0{69} $$
  The R^2^ and RMSE given by this formula are 0.8521 and 2.6359, respectively. These values indicate that the performance of the novel formula for estimating the compressive strength is satisfactory. Therefore, the proposed formula can be used in practical projects to predict the compressive strength of CGCLAC.
4. A formula describing the relationship between the splitting tensile strength and compressive strength specified in different national codes was established. The formulas in the GB50010-2010 and CEB-FIB specifications can satisfactorily predict the splitting tensile strength of CGCLAC.
5. The 28-day flexural strength of CGCLAC is between 3.3 and 5.5 MPa, and the flexural strength is approximately one tenth of the cube compressive strength. In the flexural strength test, the CGC was directly damaged at the fracture surface, unlike ordinary concrete.
6. The elastic modulus of CGCLAC is 25–35% lower than that of ordinary concrete with the same strength. The calculation formula proposed by ACI318-11 is more in line with the actual elastic modulus, and can be used to calculate the elastic modulus of ceramsite concrete.

## Data Availability

The datasets used and/or analysed during the current study available from the corresponding author on reasonable request.
